# Olfactory receptor neuron coding in the turbulent realm

**DOI:** 10.1186/1471-2202-14-S1-P315

**Published:** 2013-07-08

**Authors:** Jean-Baptiste Masson, Christelle Monsempes, Jean-Pierre Rospars, Philippe Lucas

**Affiliations:** 1 Physics of Biological Systems, Institut Pasteur, CNRS URA 2171, France; 2 Physiologie de l'Insecte, Signalisation et Communication, UMR1272, INRA, UPMC, France

## 

Insects are able to find their mates from the sparse pheromone patches transported by turbulent air streams. This source-finding ability is remarkable because the pheromone patches don't point towards the source and because the temporal and concentration statistics encountered during the search are highly irregular. The knowledge of the coding dynamics at the single ORN level is presently incomplete and it seems mandatory to decipher these dynamics before further investigating the integration of the signal at higher levels of the olfactory system.

A receptor that would attempt to infer the distance of a source from the detection dynamics of the pheromone patches would obviously benefit from the complete evolution of the pheromone concentration with time. Yet, it is possible to build a Bayesian model based on the known dynamics of the plume propagation [1] and to infer the distance from the temporal dynamics only. Hence, an interesting question is how much of the temporal dynamics of the patches can be encoded in the ORN signals? For example, the uncorrelated statistics of patch duration is sufficient for inferring the distance, yet including the correlations between the durations of detections and non-detections will greatly improve the decoding rate [2].

As a simplified framework, we investigated the dynamical olfactory coding of ORNs of a moth, *Agrotis ipsilon*, stimulated by puffs of pheromone (*cis*-7-dodecenyl acetate) of variable duration delivered at constant concentration. The spiking responses of ORNs to single puffs varying over four decades of durations (1ms-10s) were recorded and decoded. In the absence of precise knowledge on the decoding mechanism inside the antennal lobe and in higher neural structures of the insect brain, 3 decoding schemes were utilized, based on logistic regression, support vector machine and signal dependent Poisson firing model designed to include bursting dynamics. With this protocol the relative selectivity of ORNs coding with the variation of puff duration and thus the relative information transfer could be analyzed. Then, in order to investigate the possible coding dependency of the currently detected pheromone patch on previous patch detections, double puffs coding were investigated, similarly over four decades of durations. The evolution of the ORN coding showed that the detection of previous puffs influenced the coding of the current puff and hence that instantaneous decoding could bring information on the previous puff duration and timing. Furthermore, we quantified the relative (conditional) sensitivity of the instantaneous coding with respect to the duration and spacing of the previous puff. Interestingly, we also show that the "random spiking" dynamics bears a non-negligible amount of information on the past patch dynamics detection.

The coding and decoding procedures were then evaluated on signals mimicking the temporal dynamics of patches in a turbulent stream. We found that training based on one patch and two patches allowed efficient and reliable decoding of patch dynamics in turbulent air streams. Finally, we quantified the amount of information encoded by ORNs about the duration, timing, and most importantly waiting time between consecutive pheromone puffs.
